# Valuation of Medical Innovation Handling with Uncertainty and Risk

**DOI:** 10.3390/jmahp12030016

**Published:** 2024-08-01

**Authors:** Mark Nuijten, Stefano Capri

**Affiliations:** 1 A2M, 1546 Lg Amsterdam, The Netherlands; 2 Department of Biotechnology Engineering, Ben-Gurion University, Beersheba 8410501, Israel; 3 School of Economics and Management, Cattaneo-LIUC University, 21053 Castellanza, Italy; scapri@liuc.it

**Keywords:** economic valuation, orphan drugs, uncertainty, R&D innovative drugs

## Abstract

**Background:** The purpose of this paper is to address how to handle uncertainty when performing an economic valuation of a medical innovation R&D project in orphan diseases from the perspective of the investor. **Methods:** We describe the specific uncertainty related to cash flows and the cost of capital for innovation in orphan diseases. The uncertainty in cash flows relates to sales, manufacturing and R&D costs, and probabilities of failure for each phase in the clinical trial program. We consider different net present values (NPVs) and higher standard deviations for orphan drugs compared to non-orphan drugs. **Results:** Numerical case base examples showed the differences in trade-off by an investor for R&D projects with differences in NPV and level of uncertainty. The investor will transfer the additional uncertainty in cash flows in a higher cost of capital. An alternative approach is the application of an “acceptability curve” based on a probabilistic sensitivity analysis, which displays the cumulative probabilities at a range of different values for the NPV. Finally, we consider uncertainty in the cost of capital itself by applying the Capital Asset Pricing Model (CAPM). **Conclusions:** In this paper, we described various types of uncertainty and explored various approaches to how to handle uncertainty in the economic valuation of medical innovation in orphan diseases. The bridging of health economics with economic valuation theory in the healthcare market is to our knowledge a novel approach for the valuation of medical innovation by investors.

## 1. Introduction

The economic value of a medical innovation R&D project from an investor’s perspective is a function of its current and potential future revenues (sales) and expenditures (costs). The common approach for economical valuation is applying the discounted cash flow (DCF) concept [1]. Discounted cash flow is based on the present value equation, which calculates the time value of money and compounding returns.

NPV = CF_1_/((1 + r)^1^ + CF_2_/((1 + r)^2^ + … + CF_n_/((1 + r)^n^ (1)

where:

NPV = net present value;
CF = (free) cash flow;
n = the time in years before the future cash flow occurs;
r = cost of capital.

Free cash flows from operations reflect the revenues from drug sales, and the costs of R&D, production, and marketing. The cost of capital is the minimum rate that an investor is expecting to earn when he or she invests in an R&D project, which could be considered 9% and 12% for pharmaceutical companies and biotechnology companies, respectively [1,2]. The cost of capital for the investor is based on the expectations at the time of the investment decision, rather than the expectations at the time of the reimbursement application [2]. The appropriate time horizon is 20 years, where year 1 corresponds with patent registration and year 20 represents the last year of the patent period. We assume that an innovative new drug obtains registration at year 8 and that the drug will be reimbursed within 1 year, leaving 11 years for actual sales before the patent expires. The R&D costs of the failed R&D programs should be considered according to economic valuation theory, and therefore, the allocation of the costs of R&D failures to successful R&D programs leading to EMEA or FDA approval is an important stating point in the valuation. Therefore, the probabilities of failure for each of the clinical trial phases (phases I, I, III) lower the potential cash flows expected from an investment [2,3].

The discounted cash flow method is using a deterministic approach, which is fully based on average parameter values. Hence, the NPV does not capture the spread in the statistical distributions of the parameters. The purpose of this paper is to address how to handle uncertainty when performing an economic valuation of a medical innovation R&D project from the investor’s perspective. We especially consider the valuation of innovative R&D projects for orphan diseases (e.g., biologics, immunotherapy, or gene therapy) because of the high level of uncertainty for the investor, which allows us to capture most types of uncertainty for illustration of the concepts. We exclude valuation of precision medicines, which requires capturing specific issues of uncertainty, which is beyond the scope of this paper.

## 2. Materials and Methods

### 2.1. Uncertainty in Economic Valuation

#### 2.1.1. Costs

The investor’s perspective provides an argument not to use actual costs for research and development, as there are no actual costs available yet for the investor, who will rely on forecasts based on other sources. The benchmark for forecasting the R&D costs may be the data from the literature, which are average R&D costs with a high standard deviation (SD), as the total costs vary from USD 92 million to USD 883. Hence, the investor may use these average values and apply an SD to these costs. The next step is to fine-tune these average R&D costs to a specific orphan drug. Recruitment of the necessary number of patients may need more time and effort and, as such, becomes more expensive, as well as requiring a longer follow-up of patients to obtain sufficient clinical evidence [4].

The R&D costs for a new first-in-class innovative drug for treatment of a specific disease may be higher, as there is not yet an existing infrastructure for setting up and no experience in carrying out clinical trials efficiently [1]. There are also arguments that R&D costs may be lower because registration authorities may accept trials with lower sample sizes and may not require phase III trials. This uncertainty in the actual direction of change in costs, an increase or decrease, also leads to a higher SDs

The manufacturing process for innovative break-through drugs may also be much more expensive than for traditional new drugs with a similar mechanism of action. Manufacturing costs for immunotherapy and gene therapy are much higher because of the procedure, which is more time intensive and which also requires specific expensive equipment and materials not comparable with production of traditional medicinal products (e.g., antibiotics, antidepressants), as these drugs have a completely different mechanism of action. The production process, especially of immunotherapy, is also risky, which means that immunotherapy production may fail, and repeated processes may be necessary for delivery of one single immunotherapy [5]. These higher production costs and the associated higher SDs have impact on R&D costs and operational costs after launch.

The marketing costs for a first-in-class new innovative drug may be often higher due to extra costs for establishing relationships with multiple stakeholders, especially the clinical investigators. Important technological challenges for innovative therapies, especially for immunotherapy, may lead to higher failure rates as science advances [6]. Probabilities of failure rates seem to have increased over time and are different between stages of clinical development [7]. This may result from a combination of different reasons. First, regulators are becoming more risk averse and may be more hesitant to approve innovative drugs. Second, the challenges of R&D have become larger, which require new drugs with novel mechanisms of action and with less-defined clinical endpoints. Therefore, especially for the new innovative concept of immunotherapy, there are arguments for the investor to include higher failure rates.

Hence, the uncertainty in estimates for average values applies to R&D costs, manufacturing and marketing costs, and failure rates of trials, leading to higher SDs. Hence, the uncertainty for the investor in innovative drugs includes the average values and their SDs.

#### 2.1.2. Sales

Future sales are a product of volume units, unit price, the probability of registration, reimbursement, and expected competitor’s market share [8]. The volume units are a function of the following set of parameters: population size and population growth, incidence and prevalence of disease, and proportion of patients fulfilling the criteria for the indication of the new drug. Their average values and standard deviations can be estimated based on specific data for the orphan drug, but these values do not depend on the type of innovation. However, the subsequent parameters, which narrow the patient population size, do depend on the type of innovation: proportion of eligible patients, risk of prescription restriction (e.g., due to high budget impact or sufficient effectiveness/cost-effectiveness only in subpopulations), off-label use (within or outside registered indication), expected uptake curves, and probability of reimbursement. The expected competitor’s market share is based on other potential innovative drugs in clinical development for similar indications which may enter the market in future.

The clinical development of orphan drugs is characterized by a low sample size and heterogeneity in efficacy, which leads to risk of not reaching statistically significant meaningful clinical outcomes (no registration, no reimbursement) and also to a huge spread in cost-effectiveness outcomes (registration, but no reimbursement), consequently leading to higher SDs for these innovation-specific parameters than for non-orphan drugs. In addition, prices of orphan drugs ought to be higher than prices of non-orphan drugs to compensate the risk of lower sales in the small population sizes for orphan drugs.

This uncertainty also includes the risk that health authorities will not consider the primary clinical outcomes clinically meaningful, even if statistically significant and accepted for registration. Health technology assessment (HTA) bodies usually criticize the clinical effectiveness of orphan drugs because of (1) no relevant clinical outcomes; (2) uncertainty in defining an appropriate minimal clinically important difference; or (3) no validated outcome measures [9]. Another concern is that there is no correlation between surrogate and clinical outcomes. However, the heterogeneity does not allow the use of more clinically relevant outcomes, as clinical trials would require an unrealistic sample size and follow-up time to show any significant difference in clinical benefit.

The probability of a positive reimbursement decision by national health authorities not only depends on the registration data for efficacy and safety but also additional critical determinants, e.g., cost-effectiveness and budget impact, and their weight varies per country [10]. For example, the incremental cost-effectiveness ratio (ICER) is the additional cost for a life year gained in perfect health, or quality-adjusted life year (QALY) [11]. This ICER is considered critical for a reimbursement assessment in the United Kingdom, whereas budget impact is currently more important in France, Italy, and Germany. The probability of reimbursement for innovative therapies may be lower because the huge ICERs lower the likelihood that the new drug will be cost-effective at a country-specific threshold.

Uncertainty in uptake curves depends on changes in clinical guidelines, the expected entry of competitive products following registration of a new drug which may both lower volume units and prices due to competition, and other potential determinants (e.g., expected generic substitutes, changes in pricing, and reimbursement legislation). Historical data on diffusion curves for previous innovative drugs with the same indication may be used. However, if the new innovative drug has significantly different clinical properties and price (e.g., substantially higher), these historical uptake curves may not be valid for the new drug. Hence, uptake curves for immunotherapy or gene therapy may be lower, with an additional uncertainty (higher SDs). However, the increasing future available prescription data for highly priced orphan drugs may facilitate the development of uptake curves and reduce uncertainty.

The pricing potential of innovative drugs is constrained by various policies used by decision makers. A systematic literature review concluded that the most common dimension of innovation identified was therapeutic benefit, and another conclusion was that there is internationally a lack of a unified definition of innovation among regulatory authorities and health technology assessment bodies [12].

In countries where the ICER is the critical determinant, value-based pricing can be applied based on the ICER. The health authorities usually start the price negotiations with a required price discount in many countries. In particular, when the ICER is a criterion, the confidential discount should lower the ICER to the ICER threshold [3]. For example, the Dutch health authorities requested a discount of 80% for Orkambi, a biological drug for the treatment of cystic fibrosis with a list price of EUR 170,000. The EUR 34,000 resulting from this discount is the break-even price, where the ICER for Orkambi equals EUR 80,000 per QALY, which is the upper Dutch cost-effectiveness threshold [3]. This ICER-based, value-based pricing approach has limitations, especially for orphan drugs, because of huge spreads in the ICER, and therefore broader and alternative approaches for value-based pricing may be used: multicriteria decision analysis (MCDA), comparative effectiveness research, ‘managed entry agreements’ (MEAs), and related business models (price–volume agreements, patient access schemes, and performance-based/outcome-based models). The most applicable MEAs for orphan drugs are ‘pay-for-performance’ models, which use treatment targets for reimbursement. There is an extra risk for gene therapy that no long-term effects on lasting efficacy and safety are known at time of registration, and therefore, staggered payment arrangements linked to ‘pay for performance’ can be implemented [13]. Summarizing, it is expected that a price of more than EUR 200,000, as is common for orphan drugs, will lead to price negotiations with demands for a huge price discount and/or staggered payment arrangements. The investor may include the risk of limited pricing potential in cost of capital rather than cash flows.

The investor may also include current policy proposals for future pricing constraints, which also include additional criteria for safety, efficacy, and cost-effectiveness, like socially and economically acceptable pricing, fair pricing, and affordability for patients and health systems [14].

#### 2.1.3. Cost of Capital

A separate parameter in the discounted cash flow model is the cost of capital, which reflects the investor’s risk and the required return for the investor. The average cost of capital in the pharmaceutical market is an appropriate rate. The expectations at the time of the investment determine the cost of capital rather than the expectations at the time of the reimbursement application. A specific type of uncertainty should not be included in the cost of capital when it has been captured already in the cash flows (costs and/or sales) as this would lead to double counting.

The operational expectations ought to be included, if possible, in the numerator (free cash flows) of the present value equation (Equation (1)), and non-operational uncertainty ought to be included in the denominator (cost of capital, e.g., financial expectations) [3].

## 3. Results

### 3.1. Handling Uncertainty in Economic Valuation

#### 3.1.1. Uncertainty and Risk

Modern finance theory distinguishes two kinds of investor risk: diversifiable risk and undiversifiable risk [15]. The undiversifiable, or systematic risk, is the risk which the investor cannot eliminate by diversification of his or her portfolio of investments, for example, macro-economic risks like global recession. If the investor has no knowledge that probabilities of failure for the clinical trial phases, probability of registration and reimbursement, forecasts of costs (R&D costs, manufacturing, and marketing), and sales for a new specific R&D project are different from an average R&D project, the unknown values of these parameters correspond with statistical risks, which are diversifiable and therefore have no impact on the required rate of return for an investment. The investor may assume for the R&D project the average values with their reported standard deviations.

However, if, for example, for a new innovative drug with a new mechanism of action, there is a “known” expectation that probabilities, costs, and sales will differ from average values, the investor will include these different values in the DCF model, leading to a different NPV. The NPV for innovative drugs like immunotherapy and gene therapy is probably lower because of (1) higher probabilities of failure in R&D, registration, and reimbursement, (2) higher costs of R&D, manufacturing, and marketing, and (3) lower sales (e.g., restrictions, ‘managed entry agreements’, and huge price discounts).

In addition to different average values, these parameters (probabilities of failure, costs, and sales) probably may also have a higher spread, reflecting a higher risk of the expectations for innovative drugs, like immunotherapy and gene therapy. These higher standard deviations have no impact on the deterministic outcome of the economic evaluation, which provides a mean NPV. Therefore, you may argue that higher uncertainty due to unknown higher standard deviations is diversifiable for the investor; if the investor spreads the uncertainty over a large portfolio of R&D investments with similar NPVs, the average NPV will be approximately the mean deterministic NPV, even if the standard deviations are different between the various R&D projects. However, if the parameters in the economic valuation have a priori known higher standard deviations, the investor, who is generally risk averse, will take into account this higher standard deviation. If an investor has to choose between two projects with the same mean NPV but with different standard deviations in the NPVs, the investor prefers the project with the lowest standard deviation. The average SD for traditional drugs is already captured by the investor in the cost of capital, and therefore, for very innovative drugs like immunotherapy and gene therapy, the investor mainly considers the increase in SD in the economic valuation as an additional risk.

Case 1 in Box 1 shows that project A and B have the same NPV of EUR 100, but project A has a higher SD (EUR 10) than project B (EUR 5), and therefore, a risk-averse investor will prefer project B. If both projects have a different mean NPV, the investor has to make a trade-off between choosing a project with a higher NPV and SD or a project with a lower NPV and SD. Case 2 shows that the absolute SD is higher for project C than for project D, but that the relative SDs versus the mean NPV for both projects is similar (10%). Project C is preferred over project D because of the higher NPV (EUR 120 versus EUR 100) and same relative SD. However, case 3 shows an example where project F, with the lowest NPV, has an absolutely but also relatively lower SD compared to project E. The investor now must make a trade-off between project E with higher a NPV of EUR 20 (EUR 120 versus EUR 100) or project F with a lower relative SD (10% versus 20%).

The first approach assumes that the investor will transfer the additional uncertainty due to the higher relative standard deviation for project E into a higher cost of capital. If we assume project F is a traditional innovative drug with an average SD and a cost of capital of 12%, the investor may add a risk premium, e.g., 2% for project E, reflecting the higher SD, which lowers the NPV from EUR 120 to EUR 110. This adjustment excludes differences in SD, and now the mean NPVs can be compared and shows that project E is preferred over project F with NPVs of EUR 110 and EUR 100, respectively. The question is what would be an appropriate risk premium for a specific higher SD? The investor can add a premium based on his experience, which seems subjective. However, this is an accepted approach in the build-up method for estimation of the cost of capital, where a company-specific risk is added to the cost of capital based on a subjective assessment of the firm. Nevertheless, the transfer from SD to cost of capital may be challenging for investors, and therefore, we also present two other approaches.

Box 1 **Impact of uncertainty on risk.**

Case 1:

Project A: mean NPV = EUR 100, SD = EUR 10

Project B: mean NPV = EUR 100, SD = EUR 5

Case 2:

Project C: mean NPV = EUR 120, SD = EUR 12 (10% of mean)

Project D: mean NPV = EUR 100, SD = EUR 10 (10% of mean)

Case 3:

Project E: mean NPV = EUR 120, SD = EUR 24 (20% of mean)

Project F: mean NPV = EUR 100, SD = EUR 10 (10% of mean)

Difference: NPV = EUR 20, and SD 5%.

After adjustment:

Project E: mean NPV = EUR 110

Project F: mean NPV = EUR 100

Difference: NPV = EUR 10.

#### 3.1.2. Acceptability Curve

**Figure 1:**
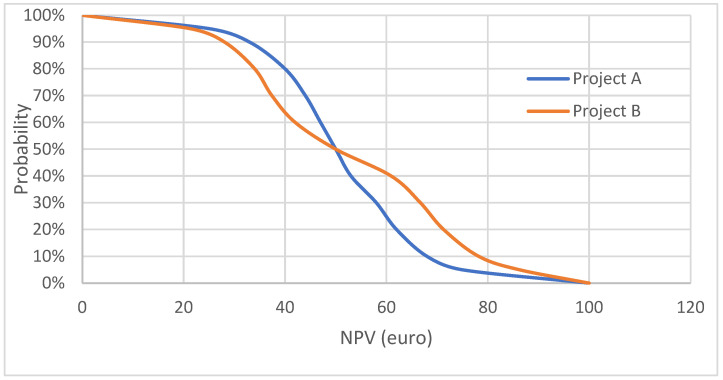
Acceptability curve—similar NPVs.

An alternative approach is application of an “acceptability curve” based on a probabilistic sensitivity analysis (PSA), which allows the inclusion of standard deviations in the input parameters of the DCF model. A PSA can explore the effect of simultaneous changes in the values of different parameters on the outcome of the DCF model, which is based on the generation of random distributions around each parameter [16,17,18]. The PSA provides the average NPV and the probability distribution of the NPV, which provides the 95% confidence interval for the NPV. The average outcome of the probabilistic analysis is approximately similar to the deterministic outcome of the DCF model, except if one or more parameters have skewed distributions. The results of the PSA can be presented graphically as a probability distribution of the NPV, which provides the 95% confidence interval. An alternative presentation is an acceptability curve, which displays the cumulative probabilities at a range of different values for the NPV (Figure 1). Project A and project B have similar 50% probability of an NPV of EUR 50, which is the average outcome of the PSA and is similar to the deterministic outcome of EUR 50. The probability is 100% for both projects that the NPV is larger than EUR 0. For Project A, there is an 80% probability of the NPV being at least EUR 40, and for Project B, there is a 65% probability of the NPV being at least EUR 40. Hence, Project A seems preferrable over Project B. However, Project A has a 25% probability of the NPV being at least EUR 60, and for Project B, there is a 40% probability of being at least EUR 70, and hence, now Project B seems preferrable over Project A. An investor with preference for risk will choose Project B, with a higher probability of a higher NPV than the average NPV of EUR 50. However, this Project B also has a higher risk that the NPV is lower than the mean NPV of EUR 50. Therefore, a risk-averse investor will choose Project A instead of Project B.

**Figure 2:**
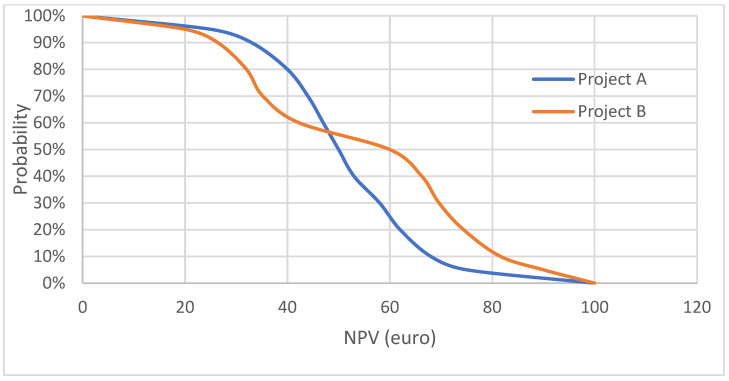
Acceptability curve—different NPVs.

Figure 2 shows different comparisons, when Project A has a different average NPV (EUR 50) than Project B (EUR 60). Project A has a 5% probability of the NPV being at least EUR 75, and for Project B, this probability is 20%. But, Project A has an 80% probability of an NPV of at least EUR 40, and for Project B, this probability is 62%. Hence, a risk-averse investor may prefer Project A, with a lower average NPV than Project B.

Hence, the use of acceptability curves allows incorporation of uncertainty by the investor for making investment decisions, which includes a trade-off of higher average NPV with higher uncertainty resulting from more spread in the input parameters but also higher uncertainty due to skewed distributions. The investor will also not select the project with the greatest area under the curve (AUC), as the AUC does not distinguish between the levels of risk, which may be different between two curves with similar AUCs but different shapes.

#### 3.1.3. Uncertainty in Cost of Capital

In the previous sections, we only considered uncertainty in cash flows and the fixed cost of capital, although the investor will transfer the additional uncertainty in cash flow to a higher cost of capital. In this section, we consider the uncertainty in the cost of capital.

The Capital Asset Pricing Model (CAPM) describes the relationship between systematic risk and expected return for assets, particularly stocks [19].

The formula for calculating the expected return of an asset given its risk is as follows:

Cost of capital == E(Ri)= Rf + βi [(E(Rm − Rf]

where:

E(Ri) = expected return of investment;
Rf = risk-free rate;
βi = beta of the investment;
E(R_m_) = expected return on market

The beta of a potential investment is a measure of how much risk the investment will add to a portfolio that looks like the market. The calculation of beta is based on the uncertainty (standard deviation) of risks and therefore the base case value of beta already captures uncertainty. Inclusion of additional standard deviation would also mean that for cash flows we would have to add uncertainty to the standard deviation. There may be an option to calculate the spread based on the distribution in beta, e.g., based on trends in the sequence of years.

The risk profile may be influenced by the therapeutic area and competitive environment and lead to a higher cost of capital for a new high-risk-profile research area like immunotherapy [13]. The investor may include a premium based on his or her expert opinion. An alternative approach is to consider incremental betas for previous new, risky comparable innovative research. Therefore, the cost of capital for R&D projects for traditional drugs may not reflect the cost of capital for new medical innovative research, e.g., immunotherapy or gene therapy. Investors therefore add a variety of premiums to the base rate. Double counting should be avoided by not including a specific risk in the cost of capital when it is already included in the cash flows. Therefore, only non-operational uncertainty, e.g., financial expectations, should be included in the premium for cost of capital [1].

The firm’s own beta is usually not a good measure of the systematic risk of one of its R&D investment projects, especially for large pharma companies. Those companies have many established drugs with reduced overall betas, whereas the beta for a risky R&D project should be higher. The company’s beta for small biotech companies with a small number of drugs in clinical development and with no actual sales may be a more appropriate estimate of beta for R&D projects.

A second consideration is that the discount rate decreases over time. Early in the R&D process, there are high fixed obligations to be met before the company can begin to earn money, so the cost of capital is higher for the money invested very early in the process than for the money invested later as the project approaches market approval. Therefore, early R&D projects are riskier than later projects and have a higher cost of capital [15]. This higher risk of early R&D is not related to the probabilities of failure in the R&D process because these are diversifiable risks. The actual rational is that operational and financial leveraging declines over the development process, which reduces the risk for the investor. This means that the cost of capital could be adjusted annually over the years until registration and subsequently a fixed annual cost of capital can be applied after market launch.

## 4. Conclusions

The common approach for economical valuation is applying the discounted cash flow concept. Discounted cash flow is based on the present value equation, which calculates the time value of money and compounding returns. The discounted cash flow method uses a deterministic approach, and hence, the economic value, e.g., the NPV, does not capture any spread in the statistical distributions of the parameters and any other non-statistical uncertainty. In this paper, we described various types of uncertainty and explored various approaches to how to handle uncertainty. Especially for innovative drugs, the level of uncertainty is higher than for traditional new generation drugs, which relates to the probabilities of failure for the clinical trial phases, costs, sales, and cost of capital. These higher standard deviations have no impact on the deterministic NP, which requires a quantification for investors. The first approach assumes that the investor will transfer the additional uncertainty to a higher cost of capital corresponding with the build-up method. Recently, Su et al. published an alternative concept to handle the uncertainty of the valuation of intellectual property [20]. A risk adjustment coefficient was calculated based on combining the AHP with set-valued statistics. Another approach is based on the use of an acceptability curve, which allows trade-offs by investors between NPV and uncertainty resulting from more spread in the input parameters.

This paper mainly captures uncertainty related to new innovations in orphan diseases without an existing indication. However, an increasing number of innovations result from known molecules, which are further developed to address healthcare needs and deliver relevant improvement for patients [21,22]. These value-added medicines (VAMs) do not require large-scale clinical trials as the evidence for market authorization. On the other hand, the health technology assessment process of VAMs remains heterogeneous across countries, and it has been1 primarily designed for originator pharmaceuticals with confirmatory evidence collected alongside pivotal clinical trials. This mismatch means that the valuation of VAMS requires specific handling of different levels of uncertainty, as presented in this paper.

## Data Availability

The original contributions presented in the study are included in the article, further inquiries can be directed to the corresponding author/s.
